# The Use of New Technologies for Improving Reading Comprehension

**DOI:** 10.3389/fpsyg.2020.00751

**Published:** 2020-04-23

**Authors:** Agnese Capodieci, Cesare Cornoldi, Elizabeth Doerr, Laura Bertolo, Barbara Carretti

**Affiliations:** ^1^Department of General Psychology, University of Padova, Padua, Italy; ^2^Azienda Sociosanitaria Ligure 5 Spezzino, La Spezia, Italy

**Keywords:** reading comprehension, training, distance rehabilitation program, digital device, Cloze app

## Abstract

Since the introduction of writing systems, reading comprehension has always been a foundation for achievement in several areas within the educational system, as well as a prerequisite for successful participation in most areas of adult life. The increased availability of technologies and web-based resources can be a really valid support, both in the educational and clinical field, to devise training activities that can also be carried out remotely. There are studies in current literature that has examined the efficacy of internet-based programs for reading comprehension for children with reading comprehension difficulties but almost none considered distance rehabilitation programs. The present paper reports data concerning a distance program *Cloze*, developed in Italy, for improving language and reading comprehension. Twenty-eight children from 3rd to 6th grade with comprehension difficulties were involved. These children completed the distance program for 15–20 min for at least three times a week for about 4 months. The program was presented separately to each child, with a degree of difficulty adapted to his/her characteristics. Text reading comprehension (assessed distinguishing between narrative and informative texts) increased after intervention. These findings have clinical and educational implications as they suggest that it is possible to promote reading comprehension with a distance individualized program, avoiding the need for the child displacements, necessary for reaching a rehabilitation center.

## Introduction

Reading comprehension is a fundamental cognitive ability for children, that supports school achievement and successively participation in most areas of adult life (Hulme and Snowling, 2011). Therefore, children with learning disabilities (LD) and special educational needs who show difficulties in text comprehension, sometimes also in association with other problems, may have an increased risk of life and school failure (Woolley, 2011). Reading comprehension is, indeed, a complex cognitive ability which involves not only linguistic (e.g., vocabulary, grammatical knowledge), but also cognitive (such as working memory, De Beni and Palladino, 2000), and metacognitive skills (both for the aspects of knowledge and control, Channa et al., 2015), and, more specifically, higher order comprehension skills such as the generation of inferences (Oakhill et al., 2003).

Recently, due to the diffusion of technology in many fields of daily life, text comprehension at school, at home during homework, and at work is based on an increasing number of digital reading devices (computers and laptops, e-books, and tablet devices) that can become a fundamental support to improve traditional reading comprehension and learning skills (e.g., inference generation).

Some authors contrasted in children with typical development the effects of the technological interface on reading comprehension vs printed texts (Kerr and Symons, 2006; Rideout et al., 2010; Mangen et al., 2013; Singer and Alexander, 2017; Delgado et al., 2018). Results were consistent and showed a worse comprehension performance in screen texts compared to printed texts for children (Mangen et al., 2013; Delgado et al., 2018) and adolescents who nonetheless showed a preference for digital texts compared to printed texts (Singer and Alexander, 2017). Regarding children with learning problems, only few studies considered the differences between printed texts and digital devices (Chen, 2009; Gonzalez, 2014; Krieger, 2017) finding no significant differences, suggesting that the use of compensative digital tools for children with a learning difficulty could be a valid alternative with respect to the traditional written texts in facilitating their academic and work performance. This conclusion is also supported by the results of a meta-analysis (Moran et al., 2008), regarding the use of digital tools and learning environments for enhancing literacy acquisition in middle school students, which demonstrates that technology can improve reading comprehension.

Different procedures and abilities are targeted in the international literature concerning computerized training programs for reading comprehension. In particular, various studies include activities promoting cognitive (e.g., vocabulary, inference making) and metacognitive (e.g., the use of strategies, comprehension monitoring, and identification of relevant parts in a text) components of reading comprehension. Table 1 reports the list of papers proposing computerized training programs with a summary of the findings encountered. Participants involved cover different ages and school grades, the majority belonging to middle school and high school. The general outcome of the studies is positive due to a significant improvement in comprehension skills after the training program with long-lasting effects also during follow-up; indeed, the majority of participants involved in training programs outperformed their peers assigned to comparison groups and maintained their improvements. Specifically, several studies (O’Reilly et al., 2004; Magliano et al., 2005; McNamara et al., 2006) used the iSTART program with adolescents and young adults. This program promotes self-explanation, prior knowledge and reading strategies to enhance understanding of descriptive scientific texts. Results demonstrated that students who followed the iSTART program received more benefits than their peers, improving self-explanation and summarization. Additionally, strategic knowledge was a relevant factor for the outcome in comprehension tasks including multiple choice questions: students who already possessed good strategic knowledge improved their accuracy when answering to bridging inference questions, whereas students with low strategic knowledge became more accurate with text-based questions. Another program, ITSS, was used with younger students (Meyer et al., 2011; Wijekumar et al., 2012, 2013, 2017), with the objective to support activities based on identifying main parts and key words in a text and classifying information in a hierarchical order. Positive outcomes were found also with such program since students who followed the ITSS program significantly improved text comprehension compared to their peers in the control group.

**TABLE 1:** Synthesis of the main results of the computerized training programs on comprehension present in the literature.

Author/s	Computerized training program	Type of control group	Trained reading comprehension components	School grade	Measures	Efficacy (during/after post-test)
**Typical Development**						
O’Reilly et al. (2004)	iSTART	Active	Cognitive (textual information, connecting text sentences) Metacognitive (self-explanation, prior knowledge, reading strategies)	End of 7th–8th	Reading Prior Knowledge Reading strategy knowledge	I > C Effects on comprehension Improvement in comprehension questions regardless of the initial strategy knowledge level
Magliano et al. (2005)	iSTART	Active (live training)	Cognitive (textual information, connecting text sentences) Metacognitive (self-explanation, prior knowledge, reading strategies)	Undergraduate	Reading Self-explanation	Skilled and less skilled readers improved with the program Less skilled readers from the live training improved more in text-based question, whereas skilled readers improved with inference questions Both treatments brought similar effects
McNamara et al. (2006)	iSTART	Active	Cognitive (textual information, connecting text sentences) Metacognitive (self-explanation, prior knowledge, reading strategies)	8th–9th	Reading strategy knowledge Self-explanation tests	I > C Effects on comprehension and self-explanation Correlation between self-explanation and comprehension questions was stronger in the intervention group
Meyer et al. (2011)	ITSS	No control (comparison between standardized or tailored version of ITSS).	Cognitive (recall specific text structure, identify key words, classification of information)	5th	Reading comprehension	Both groups improved More improvement with the individualized version
Wijekumar et al. (2012)	ITSS	Active	Cognitive (recall specific text structure, identify key words, classification of information)	4th	Reading comprehension	I > C Small but significant effects on comprehension
Wijekumar et al. (2013)	ITSS	Active	Cognitive (recall specific text structure, identify key words, classification of information)	4th–5th	Silent Reading	I > C; Small but significant effects on comprehension
Ortlieb et al. (2014)	myON^®^	3 Groups Print-based Hybrid myON	Cognitive (online reading and text analysis)	4th	Reading Comprehension	All groups improved in comprehension myON group was outperformed by the other two when using printed material myON group improved reading comprehension in the digital format
Wijekumar et al. (2017)	ITSS	Active	Cognitive (text structure recognition, hierarchical classification of information)	7th	Reading comprehension	I > C (in all measures) Small but significant effects on comprehension
**Learning Difficulties**						
Leong (1992)	DECtalk	Active	Cognitive (vocabulary, text-to-speech skills)	6th–8th	Vocabulary Reading comprehension	C > I (when combining the program with online reading explanations)
Johnson-Glenberg (2005)	3D Readers	Within groups design with control conditions	Metacognitive (comprehension monitoring, graphic organizers, question answering, question generation, summarization)	6th–7th	Comprehension assessed with open-ended answers Comprehension assessed with vocabulary gains Rereading assessed with ScrollBacks Question generation	Higher performance in comprehension and longer rereading time in the metacognitive condition
Kim et al. (2006)	CACSR	Active	Cognitive (recognition or relevant elements of the text, connecting text sentences) Metacognitive (monitoring before, during and after reading, previous knowledge)	Middle-school	Comprehension Writing main ideas from a text Decoding	I > C Positive effects on comprehension Perceived improvement from students and teachers
Potocki et al. (2013)	LoCoTex (reading comprehension, intervention group) v.s. Chassymo (decoding, control group)	Active	Cognitive (inference making, text analysis)	2nd	Word reading Non-verbal intelligence Word span; Updating Listening and reading comprehension Vocabulary; Comprehension monitoring	I > C Positive effects on reading and listening comprehension Effects on monitoring and vocabulary (significative only until follow-up)
Niedo et al. (2014)	RAP	Active	Cognitive (sentence logic, Cloze technique, paragraph understanding)	4th	Word reading Pseudoword reading Reading comprehension accuracy Silent word reading Silent sentence reading Working memory Attention and hyperactivity rating	Both groups improved Attention and working memory predicted post-test accuracy (silent reading comprehension)
Cullen et al. (2014)	Headsprout Comprehension Program	No control	Cognitive (vocabulary, text analysis, answering questions)	3rd and 5th	Reading comprehension	Substantial improvement in reading comprehension
Potocki et al. (2015)	LoCoTex vs Chassymo	4 Groups normal readers poor decoders poor comprehenders general poor readers	Cognitive (inference making, text analysis)	6th–7th	Silent word reading Fluency Listening comprehension	Improved fluency with decoding training Improvement in word recognition, listening and reading comprehension with comprehension training
Kleinsz et al. (2017)	LoCoTex vs Chassymo	3 Groups: Specific decoding difficulty Specific comprehension difficulty General reading difficulty	Cognitive (inference making, text analysis)	2nd	Written word recognition Listening and reading comprehension Decoding Phonological skills Decoding fluency Vocabulary Comprehension monitoring Working memory Non-verbal reasoning	Difficulties in working memory during testing phases in all three groups Improvements in word decoding and phonological awareness with decoding training Improvements in vocabulary, comprehension monitoring, decoding fluency and accuracy, word-recognition with comprehension training

*I > C, intervention group outperformed control group; iSTART, Interactive Strategy Training for Active Reading and Thinking; ITSS, Intelligent Tutoring for the Structure Strategy; DECtalk, Text-to-speech program; CASR, Computer-Assisted Collaborative Strategic Reading; PILE, ProgramaInformatizado de LeituraEstratégica; RAP, Rapid Accelerated-reading Program. Active control: group not involved in the experimental training but receiving some kind of treatments (for example other exercises or games).*

Although most of the literature deals with typical development, also cases of students with learning difficulties were considered. For example, Potocki et al. (2013) (see also Potocki et al., 2015) examined the effects of two different computerized programs with specific aims: one focusing on comprehension features, such as inference making and the analysis of text structure, the other considering decoding skills. Both training programs brought some benefits to reading comprehension, however larger effects were found with the program focused on comprehension with long-lasting effects in listening and reading comprehension (see also Kleinsz et al., 2017). Studies by Johnson-Glenberg (2005) and Kim et al. (2006), using respectively the programs 3D Readers and CACSR, were able to promote reading comprehension abilities in middle school students through metacognitive activities. Thanks to these programs students also became more aware of reading strategies and implemented them more successfully during text comprehension. In particular, a study by Niedo et al. (2014), obtained positive results on silent reading in a small group of children struggling with reading using the “cloze” procedure. This procedure proposes exercises in which parts of a text, typically words, are missing and participants are required to complete the text guessing what is missing.

Thus, computerized programs generally seem to improve reading comprehension skills. However, it should be noticed that, in most cases, students were trained at school, without the personalized support of a clinician taking into consideration the cognitive and psychological needs of the child. In particular, to our knowledge, no program examined the effects of an internet-based distance reading comprehension program which allows the child to be trained at home in a personalized way. A useful aspect of an internet-based distance training is that the psychologist can monitor with the application (*app*) the child’s results and activities and write him/her some motivational messages, reducing the attritions present in programs carried out at home with the only supervision of parents. Literature concerning distance trainings is still rare, however, some evidence suggests that these programs may represent a good integration to other types of intervention, usually carried out at school, in a rehabilitation center or at home (e.g., Mich et al., 2013).

Therefore, despite still preliminary, we think that it is relevant to present data about a distance program developed in Italy named Cloze (Cornoldi and Bertolo, 2013), devised for rehabilitation purposes but with potential implication also for educational contexts. *Cloze* has been developed to promote inferential abilities both at a sentence- and discourse-level using the “cloze” procedure. Several findings in the literature demonstrate that abilities, such as anticipating text parts and inference making, bring improvements in text comprehension (e.g., Yuill and Oakhill, 1988) and it has been shown that one way to promote inferential competences is to improve the ability to predict parts of the text that are missing or that follow, considering the available information: the “cloze” technique appears to be one of the most successful ways for this purpose (e.g., Greene, 2001).

In the current study the effectiveness of this training program has been tested on a clinical population who exhibited, for various reasons, difficulties in reading comprehension. Participants were 28 children (16 male and 12 female) attending a private practice for learning difficulties in the city of La Spezia, in the north-west of Italy, from 3rd to 6th school grade (5 of 3rd, 9 of 4th, 11 of 5th and 3 of 6th grade), with a mean age of children of *M* = 9.79 years (SD = 1.03). Seventeen children had a current or past speech disorder: of these children 10 also had a LD (Learning Disabilities) and one was bilingual (speech problems were not due to bilingualism). The other 11 children had a LD or important learning difficulties, and one of them had also ADHD (Attention Deficit/Hyperactivity Disorder). For the goals of the study, all these children were considered together as they all presented a severe reading comprehension difficulty as reported by parents and teachers and confirmed by the initial assessment.

All children had received a comprehensive psychological assessment (see Table 2), adapted to their particular needs and ages. In particular all children had an IQ >80 assessed with the Wechsler Intelligence Scale for Children-IV (WISC-IV; Wechsler, 2003) and did not have anxiety disorders, mood affective disorders or other developmental disorders, with the exception of the cases with language disorder and the case with ADHD. Children were not receiving any additional treatment, including medication. Written consent was obtained from the children’s parents in the context of the private practice.

**TABLE 2:** Main characteristics of the sample in terms of reading and cognitive abilities.

Task	Mean	Standard deviation
**Reading assessment**		
Text reading speed (syllables per seconds)	2.70	0.81
Text reading speed (syllables per seconds) *z* score	−0.81	0.77
Text reading errors	4.32	3.7
Words reading (syllables per seconds)	2.37	0.75
Words reading (syllables per seconds) *z* score	−0.75	1.03
Words reading errors	4.79	2.3
Non-word reading (syllables per seconds)	1.73	0.66
Non-word reading (syllables per seconds) *z* score	−0.13	1.14
Non-word reading (syllables per seconds) errors	7.04	3.13
**Cognitive assessment**		
Full Scale IQ (FSIQ)	97.61	10.24
Verbal Comprehension Index (VCI)	103.04	11.29
Perceptual Reasoning Index (PRI)	103.29	11.52
Working Memory Index (WMI)	90.56	9.80
Processing Speed Index (PSI)	92.44	11.46

## Materials and Methods

### Pre-/Post-test Assessment and Procedure of the Training

Each child started a training program through the distance rehabilitation platform Ridinet, using the Cloze app, after the assessment of learning and cognitive abilities, including comprehension assessment with two texts, one narrative and one informative (Cornoldi and Carretti, 2016; Cornoldi et al., 2017). Connection to the Ridinet web site was required in order to access to the app, three or four times a week for more or less 15/20 min. The period of use was of 3 months for 6 children and 4 months for 22 children. After this period children’s comprehension was assessed again. Additionally, some questions were asked to parents and children about the app’s utility and pleasantness. In particular, children were asked: “Do you think the program helped you improve your text comprehension skills?,” “Did you like doing this program instead of the same exercises on paper?”; and parents were asked: “Was it difficult to start the Cloze activities on days when it had to be done?,” “Compared to the beginning of the treatment, how do you currently judge the ability of your child to understand the texts?”. For all questions, except the last one, the answer had to be given on a 5-point scale with 1 = not at all, 2 = a little, 3 = enough, 4 = very, 5 = very much. For the last question the answer changed on a 4-point scale with 1 = got worse, 2 = unchanged, 3 = slightly improved, and 4 = greatly improved.

### Comprehension Tasks

Reading comprehension was assessed with two texts, the first narrative and the other informative, taken from Italian batteries for the assessment of reading (Cornoldi and Carretti, 2016; Cornoldi et al., 2017). The texts range between 226 and 455 words in length, and their length increases with school grade (in order to have texts and questions matching the degrees of expertise at different grades the batteries include a different pair of texts for each grade). Students read the text in silence at their own pace, then answer a variable number of multiple-choice questions (depending on school grade), choosing one of four possible answers. There is no time limit, and students can reread the text whenever they wish. The final score is calculated as the total number of correct answers for each text. Alpha coefficients, as reported by the manuals, range between 0.61 and 0.83. For the purposes of the study we decided to use the same two comprehension texts, at pre-test and post-test, as the procedure offered the opportunity of directly examining and showing to parents changes in comprehension and previous evidence had shown the absence of relevant retest effects with this material in a retest carried out after 3 months (Viola and Carretti, 2019).

### Distance Rehabilitation Program: Cloze

Cloze (Cornoldi and Bertolo, 2013) is an app for the promotion of text comprehension with the specific aim to recover processes of lexical and semantic inference. At each work session the child works with texts that lack words and must complete the empty spaces by choosing the correct alternative from those automatically proposed by the app, so that the text becomes congruent. The program is adaptive, as text complexity and proportion of missing words vary according to the previous level of response, and is designed for children who have weaknesses in written text comprehension, mainly due to poor skills in lexical and semantic inferential processes. The app also allows to enhance a set of language skills (phonology, syntax, semantics) which contribute to ensuring the fluidity of text and production processing. The recommended age range for the use of this program is between 7 and 14 years. In this study the semantic mode (only content words may be missing and no syntactic cues can be used for deciding between the alternatives) was proposed to 21 children and the syntactic mode (where all words may be missing) to 7 children. The mode type selected for each child depends from the performance at pre-test and diagnosis. A clinician, co-author of the present study (LB), monitored the child’s results and activities with the app and sent him/her from time to time some motivational messages. The motivational messages were typically sent once a week for congratulating with children for the work done and check with him/her possible problems emerged. Training lasted from 3 to 4 months and involved between 3 and 4 sessions of 15–20 min per week. The variation in duration depended on the decision of each individual family. In fact, children were required to use the software for about 4 months or in any case for a minimum period of 3 months (choice made by six families).

## Results

### Effects on Reading Comprehension of *Cloze* Training

All analyses were carried out with SPSS 25 (IBM Corp, 2017). A preliminary analysis found that all the examined variables met the assumptions of normality (K-S between 0.106 and 0.143, *p* > 0.05). Then, we compared the reading comprehension performance of children before and after the computerized training with *Cloze*. For this analysis, a repeated measure Analysis of Variance (ANOVA) was conducted on comprehension scores to examine the differences in the whole group of children between the scores obtained before and after the training. A significant difference was found for both comprehension texts [*F*(1,27) = 22.37, *p* < 0.001, η^2^_p_ = 0.453 and *F*(1,27) = 38.90, *p* < 0.001, η^2^_p_ = 0.599, respectively]. Possible differences between the two training modalities (semantic vs syntactic) and between different training periods (3 months vs 4 months) were then analyzed; no significant differences emerged between groups in both cases [*F*(1,27) < 1].

Secondly, to analyze the role of individual differences at pre-test, the standardized training gain score (STG; Jaeggi et al., 2011) – computed by subtracting post-test score minus pre-test score, divided by the SD of the pre-test – was calculated for the two texts comprehension. Pearson correlations were computed between the STG and the variable collected at pre-test (reading speed and errors, WISC IV – Full scale IQ, Verbal Comprehension, Perceptual Reasoning, Working Memory and Processing Speed indexes). The only significant correlation was between STG of the narrative text and Verbal Comprehension Index of the WISC-IV Scale (*r* = 0.38, *p* = 0.048). Finally, individual improvements from pre- to post-test were also confirmed considering changes in performance in terms of standard deviation in relations to norms (provided by the manual). Table 3 shows the number of children for each comprehension text who improved their performance moving from a performance at least 2 standard deviations or between 1 and 2 negative standard deviations under the mean to a performance above one negative standard deviation.

**TABLE 3:** Changes in performance in relations to norms (provided by the manual) after the training program Cloze.

	Pre-test			Post-test		
		
	*z* < −2	−2 < *z* < −1	*z* > −1	*z* < −2	−2 < *z* < −1	*z* > −1
Text 1 (narrative)	4	13	12	1	3	23
Text 2 (informative)	6	16	5	1	7	19

### Perceived Utility, Pleasantness, Parents and Child’s Improvements of *Cloze*

Results concerning the answers of parents and children about utility, pleasantness and self-perceived efficacy of the app, were also analyzed. At the first question, addressing children’s perceived improvement in comprehension skills, more than half of the sample chose the alternatives “very” or “very much” (15 “very” and 5 “very much”), only 1 child answered “a little” and the others chose “enough.” At the second question, about the pleasure of doing this kind of activity instead of pen and paper activities, all children answered “very” or “very much.” Concerning parents’ questions, at the first question about the difficulty to start the *Cloze* activity, only one parent answered “enough,” a quarter of the sample chose “a little” (seven families) and all the other 20 families chose the alternative “not at all.” At the last question about the perceived training efficacy on their child’s performance, the large majority of the families chose “slightly improved” or “greatly improved” and only three parents thought their children’s ability had remained unchanged. However, no correlations between parents and child’s perceived improvements and STG in reading comprehension were found.

## Conclusion

The present study examined the effects of the use of *Cloze*, a distance rehabilitation program focused on inference skills, for improving reading comprehension, on the basis of the hypothesis that, being inference making related to reading comprehension at different ages (e.g., Oakhill and Cain, 2012), positive effects of the training activities on reading comprehension should be found.

Concerning the efficacy of computer-assisted training programs, literature highlights that many training programs are devised for an educational context. Results are generally encouraging with positive effects on reading comprehension, measured with materials different from those practiced during the training. However, few studies analyzed the efficacy in children with specific reading comprehension problems, and no studies considered the possibility of carrying out a training at home under the distance supervision of an expert. The latter characteristics are those that make the *Cloze* peculiar compared to the existent literature. *Cloze* is indeed based on a rehabilitation online platform which allows the child to complete personalized training activities several times a week, without moving from his/her home, and concurrently enabling the clinician to monitor the child’s progress or manage activities’ characteristics. The advantage of this procedure is twofold: on one hand it increases the potential number of training sessions per week, on the other hand it permits to save the necessary time to reach the center for rehabilitation and to reduce the costs of the intervention.

The preliminary data on *Cloze* were generally positive: children, working on either two slightly different versions of the same program, showed a generalized improvement in reading comprehension tasks and, together with their families, expressed appreciation for the pleasantness and the efficacy of the program. Encouraging results emerged also from the analysis of individual improvements referring to normative scores, as reported in Table 3: most of the children’s performance migrated from a highly negative level to an average level.

It is noticeable that the efficacy of the training was assessed with materials different from those practiced during the training sessions, since reading comprehension tasks required to read a paper text and complete a series of multiple-choice questions. In future studies it would be interesting to analyze the effects of the program on skills known to be related to text comprehension, such as vocabulary or comprehension monitoring, for example. There is good reason to believe that since these variables are highly predictive of comprehension skills (and given that training in these skills sometimes improve comprehension; e.g., Beck et al., 1982; see also Hulme and Snowling, 2011), training that specifically targets comprehension might, in turn, lead to improvements in vocabulary or comprehension monitoring skills. Further studies are needed to explore this hypothesis.

A second relevant finding of the present study is the presence of a positive correlation between the gain obtained in one of the reading comprehension text (the narrative one) and the Verbal Comprehension Intelligence Quotient (VCIQ) index of the WISC-IV battery, showing that children who started with more resources in verbal intelligence achieved greater improvements in text comprehension at least with one type of text through the *Cloze*. The activities probably required to develop some kind of strategies, and for this reason students with larger verbal intellectual resources, who were presumably more able to develop new strategies, were more advantaged. Indeed, this amplification effect is usually found when training activities require the development of strategies (von Bastian and Oberauer, 2014). Such result has clinical and educational implications, inviting professionals and teachers to consider children’s starting resources and, if necessary, to combine activities conducted through distance rehabilitation programs with personal intervention sessions that could teach strategies and promote a metacognitive approach to reading comprehension. However, some limitations of the present study must be acknowledged. Firstly, study did not include a control group, therefore findings should be taken with caution, although normative data and previous results obtained with the same test offer support to the robustness of our results and the use of normative data offers a control measure of how reading comprehension skills are acquired in typically developing children without specific training, therefore functioning as a sort of passive control group. Secondly, the treated group, although characterized by a common reading comprehension difficulty, was partly heterogeneous, as children attended different grades and could have different diagnoses. Unfortunately, the limited number of subjects, with the consequence that it was not possible to form groups defined both by the grade and the diagnosis, did not permit to make analyses taking into account the grade and the diagnosis as between-subjects factors. Future studies should examine a more homogeneous population or consider a larger sample of children, giving more information about the efficacy of training in different children population. Additionally, the fact that the treatment was concluded with the post-training assessment did not offer the opportunity to further examine the procedure and maintenance effects with a follow-up. Despite the limitations, this study offers evidence concerning the efficacy of new methods, based on computer-assisted training programs that could be beneficial in training high-level skills such as comprehension and inference generation. Such tools can be extremely worthwhile for struggling readers who may need to receive further attention in mastering higher level reading comprehension.

## Data Availability Statement

The datasets generated for this study are available on request to the corresponding author.

## Ethics Statement

Ethical review and approval was not required for the study on human participants in accordance with the local legislation and institutional requirements. Written informed consent to participate in this study was provided by the participants’ legal guardian/next of kin. Written informed consent was obtained from the individual(s) for the publication of any potentially identifiable images or data included in this article.

## Author Contributions

AC, CC and BC contributed to the design and implementation of the research. LB provided the data. BC organized the database. AC performed the statistical analysis. ED did the literature research and wrote the section about the review of the literature. AC and BC wrote the other sections. CC contributed to the manuscript revision, read and approved the submitted version.

## Conflict of Interest

The authors declare that the research was conducted in the absence of any commercial or financial relationships that could be construed as a potential conflict of interest.
